# The Atg8 Family of Proteins—Modulating Shape and Functionality of Autophagic Membranes

**DOI:** 10.3389/fgene.2017.00109

**Published:** 2017-08-28

**Authors:** Iman Abdollahzadeh, Melanie Schwarten, Thomas Gensch, Dieter Willbold, Oliver H. Weiergräber

**Affiliations:** ^1^Institute of Complex Systems, Structural Biochemistry (ICS-6), Forschungszentrum Jülich Jülich, Germany; ^2^Institute of Complex Systems, Cellular Biophysics (ICS-4), Forschungszentrum Jülich Jülich, Germany; ^3^Institut für Physikalische Biologie und Biologisch-Medizinisches Forschungszentrum, Heinrich-Heine-Universität Düsseldorf Düsseldorf, Germany

**Keywords:** autophagy, aging, lipidation, membrane fusion, membrane curvature

## Abstract

Aging is a multifactorial process involving an accumulation of alterations on various organizational levels, which finally compromises viability and limits the lifespan of organisms. It is now well-established that many aspects of aging can be positively affected by (macro)autophagy, a mechanism of self-digestion found in virtually all eukaryotic cells. A comprehensive understanding of autophagy is thus expected to not only deepen our insight into the mechanisms of aging but to also open up new avenues toward increasing the healthy lifespan in humans. In this review, we focus on the Atg8 family of ubiquitin-like proteins, which play a crucial role in the autophagy process by virtue of their unique mode of reversible membrane association.

## Introduction

The term autophagy comprises a number of cellular degradation pathways which converge to the lysosomal compartment and are highly conserved in eukaryotic organisms. Besides enabling the cell to replenish energy resources and to generate substrates for anabolic reactions during periods of starvation, autophagy is also essential for the disposal of large structures such as protein aggregates, damaged organelles, and pathogens, which are not amenable to proteasomal degradation (He and Klionsky, 2009; Ravikumar et al., 2010). The most prominent autophagic pathway, macroautophagy, is characterized by the formation of double-membrane structures termed phagophores, which engulf cytoplasmic cargo and finally close to yield autophagosomes. These organelles (typically measuring several hundred nanometers in diameter) subsequently fuse with endosomes and/or lysosomes, resulting in acidification and degradation of their contents by acid hydrolases. Owing to its role in detoxification and quality control, autophagy contributes to the maintenance of cellular integrity; the latter is of particular importance in post-mitotic tissues, where damage cannot be “diluted” during cell proliferation. Autophagy thus has the potential to counteract aging-related processes, which invariably involve accumulation of damaged macromolecules and an accompanying decline in the function of cells and organelles (López-Otín et al., 2013). Importantly, autophagy has been found to undergo an age-associated decrease in diverse model organisms including mammals (Rubinsztein et al., 2011). The mechanisms underlying this observation are not entirely clear, but are likely to include both reduced expression of key autophagy components and alterations in upstream regulatory pathways (Martinez-Lopez et al., 2015). Over the past decades, a causal link between lifespan (or healthy lifespan, sometimes denoted health span) and autophagic capacity has been substantiated by ample experimental evidence. Mutants affecting autophagy-related (Atg) proteins, for instance, reduce the lifespan of yeast, worms, and flies, and tissue-specific knockouts in mice result in premature occurrence of aging-related symptoms. Conversely, caloric restriction as well as direct pharmacologic stimulation of autophagy is well-known to increase lifespan, and similar results were obtained after engineered overexpression of Atg proteins (Madeo et al., 2015; Martinez-Lopez et al., 2015). In accordance with the pleiotropic role of autophagy in eukaryotic cells, a number of mechanisms have been proposed to underlie its longevity-promoting effect (Rubinsztein et al., 2011; Madeo et al., 2015). The predominant one is thought to be general cytoprotection via removal of potentially toxic protein aggregates (aggrephagy), as evidenced by the beneficial effects of autophagy inducers in models of age-related neurodegenerative disorders. Notably, autophagy regulation is networked with apoptosis pathways in numerous respects, such that activation of autophagy (a pro-survival mechanism) will leave apoptosis suppressed as long as damage is not excessive, thus preventing unnecessary loss of cells. This mechanism includes removal of damaged mitochondria (mitophagy), which are potent triggers of apoptosis. Depending on the organ or tissue involved, autophagy may also exert beneficial metabolic effects, such as modulation of triglyceride distribution by mobilization of cytoplasmic lipid droplets (lipophagy). Finally, autophagy is found to support longevity by suppressing the development of cancer, possibly via effects on genomic stability. It is important to note, however, that excessive autophagy can be detrimental to the organism. For instance, the reduction of muscle mass in certain neuromuscular disorders, but also in non-muscular diseases like cancer and hepatic cirrhosis, has been associated with elevated autophagy (Castets et al., 2016).

**FIGURE 1:**
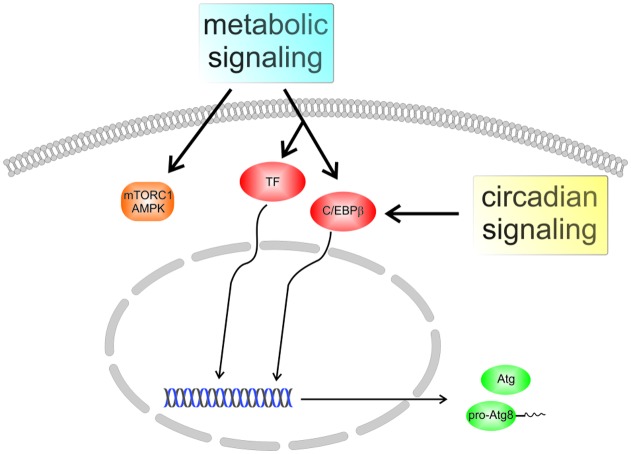
Regulation of autophagy in mammalian cells. Several pathways contribute to the adjustment of autophagic activity to the nutritional state and to stress conditions, with protein kinase complexes mechanistic target of rapamycin complex 1 (mTORC1) and AMP-activated protein kinase (AMPK) representing prominent nodes. In addition, certain autophagy-associated genes are regulated by transcription factors (TFs) like TFEB or FOXO3a, resulting in altered expression of Atg proteins. The transcription factor C/EBPβ, which was recently found to integrate metabolic and circadian signals, may play a role in the aging-related decline of autophagy.

Genetic screening in yeast has led to the identification of more than 30 *Atg* genes, most of which are conserved in mammalian cells (Nakatogawa et al., 2009). The respective polypeptides can be grouped into functional units featuring, for instance, protein or lipid kinase activities and ubiquitin-like conjugation reactions. The latter are centered on the Atg8 family of proteins which are reversibly linked to membrane lipids. Proteins of the Atg8 family were among the first components of the core autophagy machinery to be characterized in molecular detail. They are small globular molecules of about 120 amino acid residues containing an ubiquitin-like core (a so-called β-grasp fold) extended by an N-terminal helical subdomain. While the yeast *Saccharomyces cerevisiae* expresses a single Atg8, the family has expanded significantly during evolution; in humans it comprises seven active genes encoding proteins of the GABA type A receptor-associated protein (GABARAP) and microtubule-associated protein 1 light chain 3 (MAP1LC3, shortly LC3) subfamilies (Weiergräber et al., 2013). Since they are found associated with autophagic membranes at all stages of the process, Atg8 proteins are often used as markers for visualization of these structures. Given their limited size, the spectrum of biological activities attributed to these proteins is surprisingly complex, and despite intense research, their function is still not completely understood (Slobodkin and Elazar, 2013; Lee and Lee, 2016). Short-term control of the autophagy process by metabolic conditions or stress signals mostly relies on posttranslational modifications of polypeptides, with ensuing alterations to their localization and/or interaction properties. In addition, a number of *Atg* genes, such as the Atg8 family members LC3B and GABARAP-like 1 (GABARAPL1), were found to be transcriptionally regulated, involving transcription factors like TFEB, FOXO3a, or C/EBPβ (**Figure 1**). The latter not only strongly responds to nutritional signals, but also appears to be responsible for a circadian rhythm of autophagy-related mRNA levels as well as autophagic activity in mouse liver and possibly other tissues (Ma et al., 2011). Indeed, expression of C/EBPβ has recently been found to correlate with autophagy levels in fibroblasts from differently aged human donors (Kalfalah et al., 2016), suggesting this transcription factor as a potential mediator of an age-related autophagy defect. The remainder of this review is devoted to the unique characteristics of Atg8 proteins in the context of autophagy, with special emphasis on the consequences of membrane association for functionalization and morphogenesis of autophagic structures. Unless a species is stated explicitly, conserved autophagy-related proteins will be generally identified using *S. cerevisiae* notation.

## C-Terminal Lipid Conjugation

**FIGURE 2:**
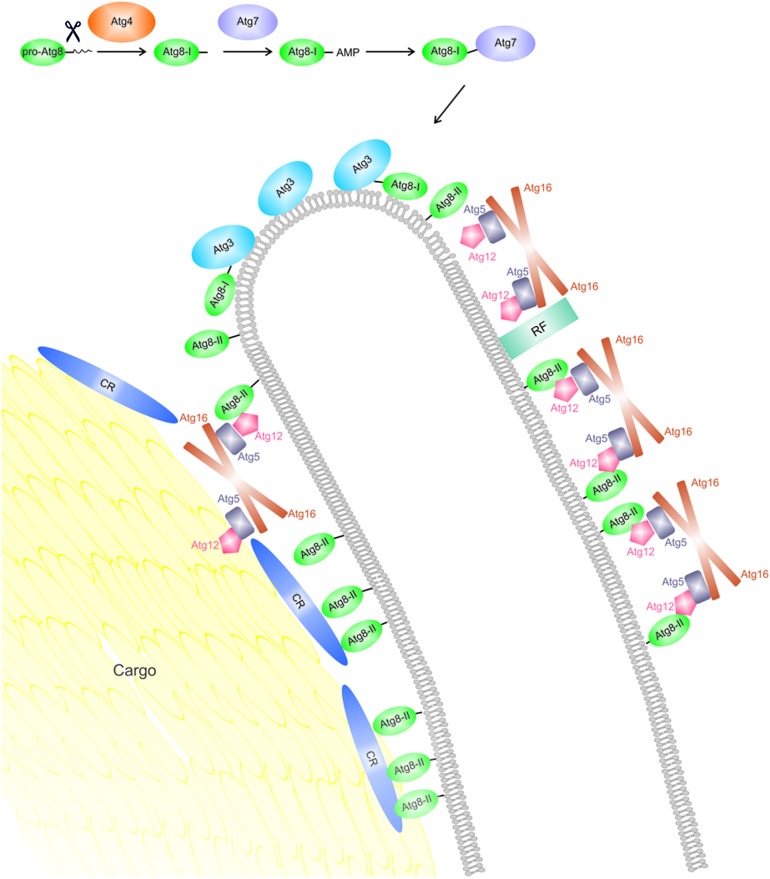
An Atg8-centric view on phagophore expansion around cargo in selective autophagy. Newly expressed Atg8 molecules (pro-Atg8) are cleaved C-terminally by Atg4, yielding a so-called form I (Atg8-I). The latter is activated by the E1-like enzyme Atg7 and transferred to the E2-like Atg3. Formation of the PE conjugate (form II, also termed Atg8-II or Atg8-PE) *in vivo* requires the Atg12–Atg5–Atg16 complex and is hypothesized to mainly occur in the rim region due to the curvature sensitivity of Atg3. Atg12–Atg5–Atg16 is recruited to the phagophore membrane by specific interaction partners; on the outer surface, these are typically PI3P binding proteins (labeled here recruiting factors, RF), while on the inner surface, cargo receptors (CRs) appear to take over this function. Lipidated Atg8 enables Atg12–Atg5–Atg16 to establish a mesh-like coat on the outer surface, which has been proposed to stabilize the shape of the organelle. On the inside, Atg8 proteins are chiefly responsible for high-avidity binding of degradation targets via cargo receptors, resulting in close apposition of the membrane.

Probably, the most well-known function of Atg8 proteins in autophagy is the recruitment of cargo molecules to be degraded in the emerging autophagosome. Specifically, they recognize short sequence motifs termed LC3 interacting regions (LIRs) or Atg8-family interacting motifs (AIMs), which are present in a number of different cargo receptors decorating the actual target structures (Rogov et al., 2014; Khaminets et al., 2016), but also in other putative interaction partners (Thielmann et al., 2009). At the same time, Atg8 proteins can be covalently but reversibly linked to the lipid phosphatidylethanolamine (PE) present in autophagic membranes, thus physically attaching the cargo to the phagophore. This reaction is accomplished via an ubiquitin-like conjugation system (**Figure 2**; Ichimura et al., 2000). The different Atg8 proteins are expressed with cleavable C-terminal extensions ranging in length from a single amino acid residue in yeast Atg8 to 21 residues in human LC3C (Ichimura et al., 2000; He et al., 2003; Kabeya et al., 2004). Specific hydrolysis yielding a glycine residue at the very C-terminus is realized by the cysteine protease Atg4. Notably, the same enzyme also catalyzes de-conjugation of Atg8 from PE at a later stage. Atg4 interacts with the Atg8 proteins via a canonical LIR at its C-terminus (Rasmussen et al., 2017). Recently, it was shown for yeast Atg4 that in addition to the LIR a so-called Atg8-PE association region (APEAR) close to the catalytic center is required for specific binding of membrane-bound Atg8 proteins, thus allowing for efficient recycling (Abreu et al., 2017). Both experimental evidence and molecular dynamics simulations indicate that the Atg8 C-terminus needs to adopt an extended conformation to access the active site of the protease (Satoo et al., 2009; Ma et al., 2015). In the next step, Atg8 gets activated by the E1-like enzyme Atg7 under consumption of ATP, yielding an adenylated intermediate which reacts to form a thioester with a cysteine residue of Atg7. The Atg8 protein is subsequently transferred to a cysteine residue of Atg3, which acts as an E2-like enzyme, and finally attached to the PE head group via an amide linkage (Ichimura et al., 2000; Tanida et al., 2003). Using a human GABARAP variant carrying a C-terminal cysteine residue for coupling to a reactive lipid, we have investigated the impact of lipidation on the structure of the protein: NMR spectroscopy revealed that the overall fold and ligand binding properties were largely unchanged with respect to the soluble counterpart (Ma et al., 2010). Nevertheless, membrane attachment plays a crucial role for most of the biological functions attributed to Atg8 family proteins, such as sequestration of substrates via cargo receptors, or recruitment of components of the autophagy machinery. In yeast cells, the overall area density of Atg8-PE on autophagic membranes has been estimated to be on the order of 1 copy per 2000 nm^2^ (Xie et al., 2009). It is worth noting that the lipidation reaction appears to be highly membrane curvature sensitive, at least *in vitro* (see below). In the cellular environment, lipidation of Atg8 proteins requires Atg12–Atg5, a second ubiquitin-like conjugate (Mizushima et al., 2001; Suzuki et al., 2001). The Atg12–Atg5 entity is constitutively associated with Atg16, and the resulting ternary complex acts as an E3-like component (Hanada et al., 2007; Fujita et al., 2008), facilitating the formation of Atg8-PE by enhancing the catalytic activity of Atg3 (Sakoh-Nakatogawa et al., 2013). Atg3 is thought to sequentially interact with Atg7 and Atg12–Atg5–Atg16 via overlapping segments of its central flexible region (Taherbhoy et al., 2011; Metlagel et al., 2013), and the localization of Atg8 lipidation appears to be mostly determined by phosphatidylinositol 3-phosphate (PI3P) binding proteins recruiting Atg12–Atg5–Atg16 to the PI3P rich phagophore membrane (Dooley et al., 2014). Recently, it was shown that several yeast as well as human cargo receptor proteins directly interact with Atg5 in the Atg12–Atg5–Atg16 complex and thus attract the conjugation machinery to the growing phagophore surrounding the cargo (Fracchiolla et al., 2016). While this mechanism is thought to promote generation of lipidated Atg8 proteins on the inner face of the phagophore, it is probably a transient association since the accumulating Atg8-PE will ultimately displace Atg5 from its binding sites on the cargo receptors, thus maintaining the asymmetry of the Atg12–Atg5–Atg16 distribution on the phagophore.

## Involvement in Phagophore Membrane Curvature

As outlined above, selective autophagy utilizes Atg8 protein binding sites (LIRs) on cargo receptors to tightly attach the phagophore membrane to the cargo, thus ensuring that non-targeted cellular material is not engulfed (**Figure 2**; Sawa-Makarska et al., 2014). Figuring the phagophore as a cup-shaped double-membrane structure with only some tens of nanometers of intermembrane space (Nguyen et al., 2017) implies bending of the lipid bilayer on different scales: while membrane curvature will be moderate on the convex (outer) and concave (inner) surfaces of the growing phagophore, it must be very high at the outer rim. Notably, the biological activity of Atg8 family proteins has recently turned out to be intricately related to the curvature of their host membranes. While investigating the effect of PE conjugation of Atg8 on artificial vesicles, Knorr et al. (2014) observed shape changes indicating an alteration of preferred membrane curvature. At the same time, Atg8-PE tends to be enriched in highly curved regions of the bilayer. The interplay of curvature generation and curvature sorting may stabilize the shape of the phagophore with its highly disparate local curvatures (Knorr et al., 2014). Moreover, in a reconstituted system the efficiency of the lipidation process itself is largest on lipid bilayers with high curvature (Knorr et al., 2014; Nath et al., 2014). Recent evidence indicates that this curvature sensitivity of Atg8 conjugation *in vitro* may be largely conferred by Atg3, which senses the local curvature via its N-terminal amphipathic helix. Specifically, packing defects of the lipid bilayer that exist primarily in highly curved areas are thought to allow insertion of hydrophobic residues of the N-terminal helix into the membrane interior. In the context of a living cell, the intrinsic curvature sensor of Atg3 appears to act as a modulator of function, rather than localization, of the enzyme (Nguyen et al., 2017). Efficient Atg8 lipidation *in vivo* requires the presence of the Atg12–Atg5 conjugate. Remarkably, Atg12–Atg5 itself was recently found to be recruited by lipidated Atg8 via an unconventional AIM in Atg12. In the presence of Atg16, Atg8-PE–Atg12–Atg5 complexes are organized into a two-dimensional mesh-like network of proteins on the membrane, which has been proposed to stabilize the shape of the phagophore (Kaufmann et al., 2014). It is important to note that Atg12–Atg5–Atg16 can be detected exclusively on the convex face of the growing phagophore and appears to be shed, together with the majority of Atg8, around the time of phagophore closure (Mizushima et al., 2001). In contrast, some Atg8-PE remains associated with the concave surface, in order to fulfill its role in cargo recruitment (Xie et al., 2008). The structural and functional asymmetry between the two surfaces of the phagophore is thus reflected by disparate sets of protein–protein interactions of surface-anchored Atg8 proteins, involving partners with multiple Atg8 binding sites in a characteristic arrangement: cargo receptors (on the concave inner face) or the Atg12–Atg5–Atg16 network (on the convex outer face). In fact, these two types of interactions may translate into characteristic ranges of favored curvature, thereby amplifying the spatial selectivity of the process.

The proposed view on the role of Atg8 proteins in shaping the phagophore membrane is consistent with results of a study evaluating the bending energy of lipid bilayers as a function of their curvature (Knorr et al., 2012). The authors showed that a double-membrane sheet tends to bend and transform into a double-membrane vesicle after reaching a critical size. This parameter essentially depends on the preferred curvatures (and thus the molecular composition) of the bilayer at the flat faces and at the curved rim of the sheet, which determine the energy difference between open and closed states. In addition, an asymmetry between upper and lower surfaces may favor a specific direction of curvature. Current evidence supports the notion that Atg8 proteins are capable of modulating all three properties mentioned above. Consequently, it seems reasonable to assume that cells may regulate autophagosome size by changing, e.g., the rate or spatial distribution of Atg8 conjugation or the availability of partners involved in scaffolds on either surface of the growing phagophore (Xie et al., 2008; Knorr et al., 2012).

## Membrane Tethering and Fusion

In addition to functionalization and shaping of autophagosomal membranes, Atg8 family proteins have been ascribed activities related to the interaction of membranous structures. Since its first description a decade ago, this function of Atg8 homologs has been investigated by several groups, but its physiological relevance is still controversial.

Using a conjugation system reconstituted from purified proteins, Nakatogawa et al. (2007) showed that liposomes decorated with yeast Atg8 tend to form large aggregates. Moreover, liposome mixing experiments revealed the outer leaflets of the vesicle membranes to fuse whereas the inner leaflets remained separate, indicating hemifusion of liposomes upon attachment of Atg8. These activities appeared to require protein–protein interactions between Atg8 molecules on opposing membranes. Finally, the authors provided evidence for involvement of Atg8-mediated tethering and hemifusion in the expansion of the isolation membrane (Nakatogawa et al., 2007). Weidberg et al. (2011) essentially confirmed these observations for GABARAP-like 2 (GABARAPL2) and LC3B, representing the two Atg8 subfamilies in mammalian cells, and a chemical lipidation system. Their results, however, point to full fusion of membranes rather than hemifusion; whether this discrepancy reflects protein-specific functions or is due to differences in the experimental setup has remained unclear. The authors were able to narrow down the fusion activity to the N-terminal stretches of these proteins, which are characterized by an accumulation of basic and hydrophobic residues in case of LC3B and GABARAPL2, respectively. They interpreted these data in terms of Atg8 proteins directly interacting with opposing membranes via their N-termini (Weidberg et al., 2011). The concept of Atg8 proteins mediating the fusion of small vesicles to the rim of the growing isolation membrane has been challenged by the finding that the fusogenic activity strongly depends on the lipid composition of the membranes involved; while yeast Atg8 readily induced fusion of liposomes containing 55 mol% PE, reduction to 30 mol% (a fraction claimed to be more physiological) largely eliminated this effect (Nair et al., 2011). These observations were essentially independent of the mechanism of Atg8-membrane attachment, thus ruling out the impact of PE content on lipidation efficiency. In contrast to membrane fusion, the tethering of Atg8-decorated vesicles was unaffected by their PE content. Landajuela et al. (2016) have recently re-investigated the tethering and fusion capabilities of human Atg8 proteins as well as the modulating effects of lipid composition. Irrespective of the lipidation mechanism, they observed GABARAP and GABARAPL2 to efficiently mediate clustering of liposomes, mixing of lipids in both membrane leaflets, as well as mixing of vesicular contents, indicating full fusion. In contrast, LC3 was much less efficient as a fusogen, but instead appeared to induce changes in vesicle shape, promoting more elongated structures (Landajuela et al., 2016). Intriguingly, lipids with negative spontaneous curvature like cardiolipin, diacylglycerol, or PE were found to promote vesicle fusion mediated by GABARAP or GABARAPL2, whereas the opposite effect was observed for lysophosphatidylcholine, which induces positive curvature. These results are consistent with the canonical fusion model involving a stalk, i.e., a connection between the outer leaflets with strong negative curvature. Finally, the fusogenic activity of GABARAP and GABARAPL2, but not LC3, turned out to be stimulated by a reduction in vesicle radius. This finding adds to the growing body of evidence relating the autophagy machinery to membrane curvature (discussed above) and supports the idea that the isolation membrane may expand via fusion of small vesicles to its (similarly curved) edge. It should be noted, however, that the precise lipid composition of these organelles has not been reported to date, although protocols for the enrichment of autophagosomes from cell lysates have been available for some time (Seglen and Brinchmann, 2010). In contrast to fusogenicity, the membrane tethering activity of Atg8 family proteins has been consistently observed in various experimental settings and for different lipid compositions, and is thus likely to represent a biological activity of these molecules. Notably, the recent observation that adhesion zones between Atg8-decorated vesicles display a fairly constant Atg8 density (Knorr et al., 2014) supports the concept of an ordered three-dimensional array formed at the membrane interface that is independent of the overall density of lipidated protein.

## Conclusion and Future Perspective

G. Kroemer and coworkers have defined nine hallmarks of aging suggested to represent either primary causes of damage, detrimental effects of regulatory mechanisms, or compromised tissue homeostasis resulting in functional defects (López-Otín et al., 2013). Indeed, most of these hallmarks have been directly or indirectly linked to autophagy. Given that a wealth of experimental evidence qualifies this pathway as a core anti-aging mechanism in eukaryotic cells, sustaining juvenile levels of autophagy has emerged as a promising strategy to prolong healthy lifespan in humans. Virtually all aspects of functional macroautophagy depend on the presence of Atg8 proteins, which, by virtue of their unique membrane linkage, not only recruit components of the autophagy machinery as well as cargo to the growing phagophore but also appear to modulate biophysical properties of the membrane itself. Among mammalian Atg8 family proteins, GABARAPL1 features the highest transcript levels in the central nervous system. Intriguingly, it was found to directly bind α-synuclein oligomers, at least *in vitro*, suggesting that it might be involved in clearance of these structures, which are implicated in the pathogenesis of Parkinson’s disease (Schnack et al., 2008). This function, however, would be compromised by downregulation of GABARAPL1 mRNA expression, which has been observed in *Substantia nigra* neurons of affected subjects at later stages of disease (Simunovic et al., 2009). Provided that a role of GABARAPL1 in the removal of pathogenic oligomers can be verified *in vivo*, transcriptional re-activation of its gene may represent a strategy for future pharmacological intervention in Parkinson’s disease and possibly other neurodegenerative disorders. Beyond their well-established role in autophagy, Atg8 proteins may be implicated in aging processes in additional, previously unanticipated ways. Results from our laboratory suggest that mammalian GABARAP subfamily members are required for secretion processes generating extracellular vesicles (Boeske et al., 2017). If produced by challenged cells, such vesicles may carry altered macromolecules serving as damage-associated molecular patterns (DAMPs), or signaling molecules such as microRNAs, possibly contributing to a chronic low-grade auto-inflammatory state characteristic of organismal aging (Franceschi et al., 2017) as well as cell-to-cell propagation of a senescent phenotype (Eitan et al., 2016). Future studies will need to address the molecular details underlying the role of Atg8 proteins in secretion and their significance for the progression of aging.

## Author Contributions

All authors listed have made a substantial, direct and intellectual contribution to the work, and approved it for publication.

## Conflict of Interest Statement

The authors declare that the research was conducted in the absence of any commercial or financial relationships that could be construed as a potential conflict of interest.
